# Assessment of chronic allograft injury in renal transplantation using diffusional kurtosis imaging

**DOI:** 10.1186/s12880-021-00595-3

**Published:** 2021-04-07

**Authors:** Xin Zheng, Min Li, Pan Wang, Xiangnan Li, Qiang Zhang, Song Zeng, Tao Jiang, Xiaopeng Hu

**Affiliations:** 1 Department of Urology, Beijing Youan Hospital, Capital Medical University, No. 8, Xi Tou Tiao, Youanmen Wai, Fengtai District, Beijing, 100069 People’s Republic of China; 2 Department of Radiology, Beijing Chao-Yang Hospital, Capital Medical University, 8 Gongren Tiyuchang Nanlu, Chaoyang District, Beijing, 200020 People’s Republic of China; 3 Institute of Urology, Capital Medical University, 8 Gongren Tiyuchang Nanlu, Chaoyang District, Beijing, 200020 People’s Republic of China; 4 Department of Urology, Beijing Chao-Yang Hospital, Capital Medical University, 8 Gongren Tiyuchang Nanlu, Chaoyang District, Beijing, 200020 People’s Republic of China

**Keywords:** Diffusion-MRI, Non-Gaussian diffusion, DKI, Renal transplantation

## Abstract

**Background:**

Chronic allograft injury (CAI) is a significant reason for which many grafts were lost. The study was conducted to assess the usefulness of diffusional kurtosis imaging (DKI) technology in the non-invasive assessment of CAI.

**Methods:**

Between February 2019 and October 2019, 110 renal allograft recipients were included to analyze relevant DKI parameters. According to estimated glomerular filtration rate (eGFR) (mL/min/ 1.73 m^2^) level, they were divided to 3 groups: group 1, eGFR ≥ 60 (n = 10); group 2, eGFR 30–60 (n = 69); group 3, eGFR < 30 (n = 31). We performed DKI on a clinical 3T magnetic resonance imaging system. We measured the area of interest to determine the mean kurtosis (MK), mean diffusivity (MD), and apparent diffusion coefficient (ADC) of the renal cortex and medulla. We performed a Pearson correlation analysis to determine the relationship between eGFR and the DKI parameters. We used the receiver operating characteristic curve to estimate the predicted values of DKI parameters in the CAI evaluation. We randomly selected five patients from group 2 for biopsy to confirm CAI.

**Results:**

With the increase of creatinine, ADC, and MD of the cortex and medulla decrease, MK of the cortex and medulla gradually increase. Among the three different eGFR groups, significant differences were found in cortical and medullary MK (*P* = 0.039, *P* < 0.001, *P* < 0.001, respectively). Cortical and medullary ADC and MD are negatively correlated with eGFR (r = − 0.49, − 0.44, − 0.57, − 0.57, respectively; *P* < 0.001), while cortical and medullary MK are positively correlated with eGFR (r = 0.42, 0.38; *P* < 0.001). When 0.491 was set as the cutoff value, MK's CAI assessment showed 87% sensitivity and 100% specificity. All five patients randomly selected for biopsy from the second group confirmed glomerulosclerosis and tubular atrophy/interstitial fibrosis.

**Conclusion:**

The DKI technique is related to eGFR as allograft injury progresses and is expected to become a potential non-invasive method for evaluating CAI.

**Supplementary Information:**

The online version contains supplementary material available at 10.1186/s12880-021-00595-3.

## Background

For patients with end-stage renal disease, kidney transplantation may be the best treatment option. When the dialysis method is different, kidney transplantation can provide unparalleled results, such as survival rate, personal satisfaction, quality of life, and cost suitability [1].

Despite advances in surgical methods and immunosuppressants, the long-term effects of kidney allografts have not changed significantly in the last two decades. Chronic allograft injury (CAI) is the most common cause of kidney allograft failure, which can lead to a certain degree of delay.

The characteristics of CAI are glomerulosclerosis, tubular atrophy, vascular occlusive changes, and interstitial fibrosis. Early detection and exact CAI evaluation are critical to manage treatment and postpone or prevent irreversible damage to the transplanted renal [2, 3].

Current methods for evaluating CAI have significant impediments. The most widely used techniques for testing allograft function are serum creatinine levels and estimated glomerular filtration rate (eGFR). However, they are believed to be affected by many factors, and their prognostic value for allograft injury is poor. When serum creatinine levels rise or eGFR falls, allograft damage may have progressed to the point that it is no longer reversible [4]. Accordingly, allograft biopsy was considered the golden choice to analyze allograft injury and differentiate among the different etiologies, despite its impediments, such as infection, bleeding, and even allograft loss [5]. However, the regular evaluation of CAI still can not be exactly. Like the eGFR might not be sensitive enough to evaluate CAI change, and the standard golden biopsy also seems to have significant risks. Hence, a critical need is needed to find non-invasive and precise techniques for diagnosing CAI to guide timely intervention.

The morpholoy, microstructue, and functional characteristics of renal allografts have been confirmed using magnetic resonance imaging (MRI). Several pieces of research have found a significant correlation between MRI and eGFR, like diffusion-weighted imaging (DWI), diffusion tensor imaging (DTI), blood-oxygen-level-dependent (BOLD) imaging, and arterial spin labeling (ASL), which indicated the potential of using MRI as a non-invasive biomarker [6–10]. MRI does not use ionizing radiation, allowing for repeated imaging after the patient has accepted a kidney transplant [7, 11].

Diffusional kurtosis imaging (DKI) is an extension of the traditional diffusional kurtosis imaging (DTI) model that takes into account non-microstructural complexity and diffusional heterogeneity. DKI needs at least three b-values, with the highest b-value exceeding DWI. Although standard DTI metrics like mean diffusivity (MD) and apparent diffusion coefficient (ADC) are available, DKI can provide parameters defined by mean kurtosis (MK). Among these parameters, MK is the significant one obtained from DKI, which renal allografts are believed to be a natural complexity. The degree of diffusion restriction on the non-Gaussian distribution of water molecules increases with the complexity of the structure and the MK value [12]. As a result, we hypothesized that the DKI technique could be associated with eGFR and could show microstructural changes in CAI more specifically than eGFR, and that the DKI technique could be a non-invasive tool for assessing CAI.

This study is planned to evaluate the correlation between eGFR and DKI parameters and the potential feasibility of the DKI technique in non-invasive assessment for CAI.

## Methods

### Participants

All the patients included in the analysis received kidney transplantation approved by the Institutional Review Board of Beijing Chao-Yang Hospital, Capital Medical University. They conformed to the tenets of the Declaration of Helsinki. Before allograft biopsy and functional MRI, written informed consent from all patients should be collected.

In this prospective single-center study conducted from February 2019 to October 2019, a total of 130 adult patients after kidney transplantation agreed to participate and had no MRI contraindications were inrolled. The existence of MRI-incompatible devices, claustrophobia, patient rejection, and a time span of less than three months between transplantation and MRI were all conditions for exclusion. When the MRI slot is available, select eligible patients who did not meet the exclusion criteria. The final analysis included only kidneys known to have been procured with the donors' consent or family members. The final report did not include data from patients who had kidney transplants from unknown sources.

Within one week of the biopsy, functional MRI examinations were performed. Blood samples were taken on the day of the MR test from all patients, and eGFR was determined by changing the renal disease equation [13]. Patients were divided into three groups based on allograft function: Group 1 had patients with appropriate allograft function (eGFR > 60 (mL/min/1.73 m^2^), Group 2 had patients with mild allograft function (30 (mL/min/1.73 m^2^), and Group 3 had patients with severely impaired renal function (eGFR30 (mL/min/1.73 m^2^).. Clinical status and renal function were monitored every 1–4 weeks in the first year after transplantation and every month after that. A minimum of 3 months of follow-up was available for all patients.

### MRI protocols

All MRI examinations were performed on the 3T Prisma MRI scanner (Siemens Healthineers, Erlangen, Germany) using a 16-channel phased-array coil positioned over the pelvis. The anatomic images were obtained using transverse T1-weighted imaging (TR/TE = 700/12 ms, slice thickness = 5.0 mm, slice spacing = 0 mm, FOV = 240mm^2^, matrix size = 256 × 256) and transverse T2-weighted imaging (TR/TE = 3770/101 ms, slice thickness = 5.0 mm, slice spacing = 0 mm, FOV = 240mm^2^, matrix size = 320 × 320) covering the transplant kidney. Following the anatomic scans, DWI was performed with single-shot echo-planar imaging (EPI) in the transverse plane, with locations identical to those prescribed for the transverse T2-weighted imaging, using six *b*-values (0_4_, 400_6_, 800_8_, 1200_10_, 2000_10,_ and 3000_12_ s/mm^2^, where the subscript denotes the number of averages). The other key acquisition parameter for DWI were: TR/TE = 3100/68 ms, slice thickness = 3.5 mm, slice spacing = 0 mm, matrix size = 120 × 120, FOV = 350 mm, and the scan time ~ 9 min.

### Functional MRI image analyses

All MR images were analyzed by two radiologists (with more than ten years of experience in abdominal imaging) who did not know the diagnosis or pathophysiologic grade. Regions of interest (ROI) were manually drawn on DWI with *b* value = 0 s/mm^2^ and then copied onto the corresponding position on all parameter maps. 18 ROIs were positioned on three parts (upper pole, central area, and lower pole) of the transplanted kidney: for each segment, three ROIs (anterior labrum, posterior labrum and, intermediate site) were drawn on the cortex and another three on the medulla. Finally, the values of ADC and DKI parameters (MK and MD) were calculated as an average of all voxels contributing to the ROI, and then did further quantitative analysis detailed below. Artifacts were avoided when placing ROIs. All measurements were repeated twice, and the values of K, D, and ADC were recorded.

### Histopathological analysis

We performed biopsies randomly in patients of Group 2 to confirm the histopathological changes. The biopsy indicated eGFP < 60 (mL/min/1.73 m^2^) with or without gradual deterioration during the follow-up and patient consent. Kidney tissue was obtained by needle biopsy and fixed in 4% buffered formaldehyde. The tissue was then embedded in paraffin and serially sliced into 2-m thick portions. To evaluate glomerulosclerosis and tubular atrophy, the parts were stained with hematoxylin and eosin (H&E) and periodic acid silver methenamine (PASM), respectively, and Masson-trichrome Goldner's to measure interstitial fibrosis. The diagnosis procedures were performed according to the renal pathology laboratory's standard protocols, as reported previously [14]. Two qualified neuropathologists with 15 years and 18 years of experience, respectively, conductedd pathologic diagnoses according to the Banff 2015 scheme [15] without referring to functional MRI results.

### The inter-reader agreement assessment

We evaluated the repeatability of MRI parameters using interclass correlation coefficients(ICCs). The ICC was classified as good consistency when it was larger than 0.80.

### Statistical analysis

SPSS 24.0 for Windows (SPSS, Chicago, IL, USA) was used for statistical data analysis. We used the Kolmogorov–Smirnov test to testify the normality of the data. Variables that are normally distributed are represented as mean values with standard deviations. Categorical variables are described as a percentage. We used Two-tailed paired Student *t*-tests to compare cortical and medullary DKI parameters. A one-way analysis of variance (ANOVA) was performed to test the difference in ADC, Mean K, and Mean D values between the three groups. A Pearson correlation analysis was performed to determine the relationship between the MRI parameters and eGFR. The receiver operating characteristic (ROC) curve analysis of diffusional kurtosis parameters was performed to predict normal and mildly impaired renal function in the recipients to evaluate the diagnostic performances of diffusional kurtosis parameters in predicting impaired renal function without the server impaired renal function group. *P* < 0.05 indicated that the difference was statistically significant.

## Results

### Patient demographics, clinical characteristics, and laboratory characteristics

**Fig. 1:**
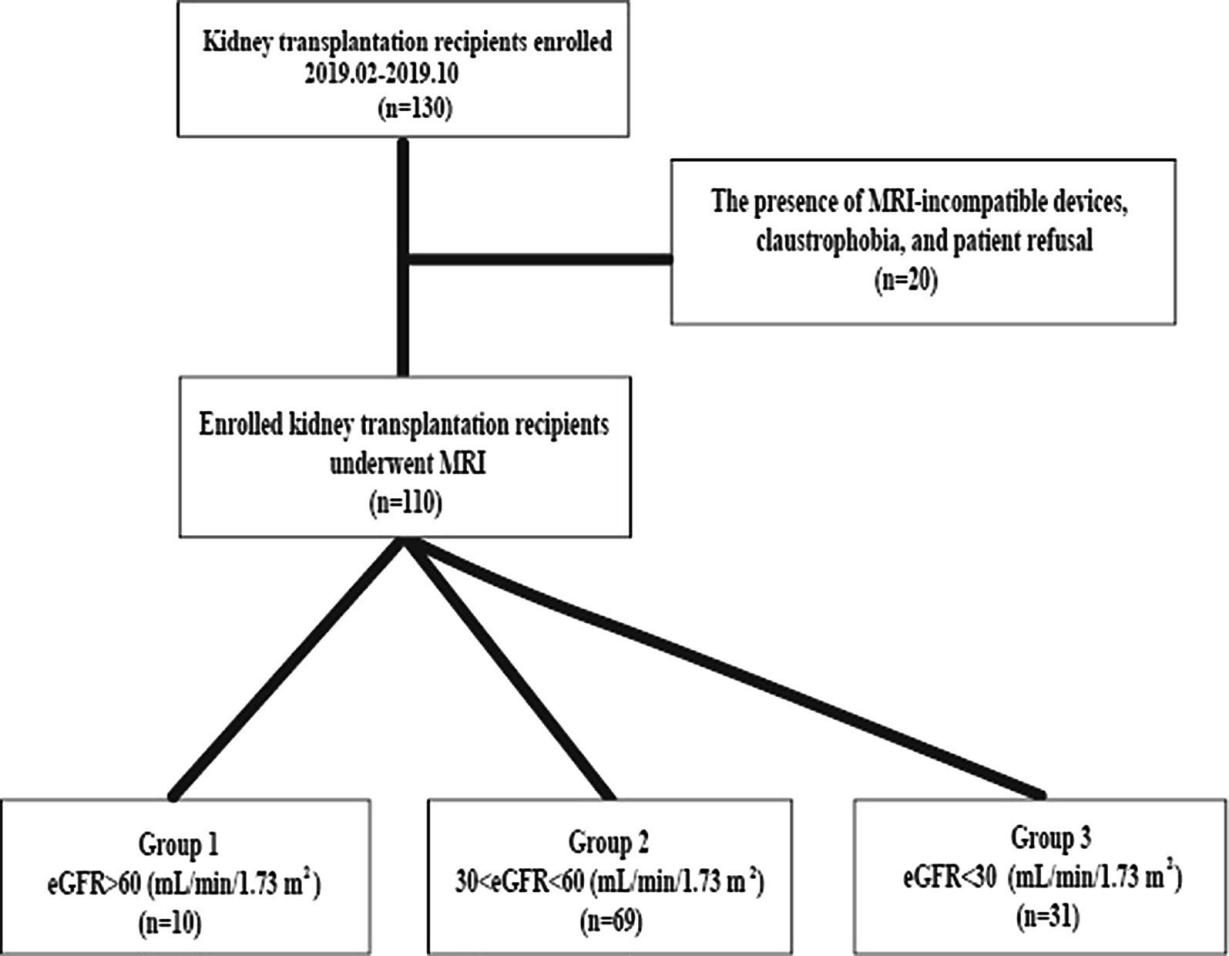
Flowchart of patient enrollment, exclusion criteria, final study sample, and categorization of patients to different groups

A total of 130 patients were included in this cohort, and if there were MRI-incompatible devices, claustrophobia, and patient refusal(n = 20), they were excluded. Finally, the functional MRI data of 110 patients (mean age, 44.32 ± 9.562 years) were analyzed (Fig. 1), containing 82 males (mean age, 43.50 ± 9.535 years) and 28 females (mean age, 46.71 ± 9.40 years). The time interval between renal transplantation and MR examination was three months to 300 months, with a median of 42.29 months. No statistically significant difference was observed between these three groups about donor demographics, recipient age, sex, kidney transplant type, and immunosuppressive regimens used. (Table 1).

**Table 1:** Patient demographics, clinical features, and laboratory characteristics in 110 patients who underwent functional magnetic resonance imaging

Characteristics/findings	Group 1	Group 2	Group 3
No. of patients	10	69	31
Sex (men: women)	5:5	11:58	12:19
Age(months)	50.4 ± 5.4	43.1 ± 9.5	44.9 ± 10.3
Time post transplantation(months)	35 ± 25.8	31.96 ± 26.1	67.32 ± 47.2
*Immunosuppressive regimens*			
Pre + MMF + FK506, n (%)	8(80)	60(87)	27(87.1)
Pre + MMF + CsA, n (%)	0	6(8.7)	2(6.5)
Other, n (%)	2(20)	3(4.3)	2(6.5)
Serum creatinine, mg/dl, mean ± SD	65.3 ± 4.6	108.8 ± 33.6	168.4 ± 104.6
eGFR, ml/min/1.73 m^2^, mean ± SD	65.6 ± 5.1	41.7 ± 8.8	24.3 ± 8.1
Hemoglobin level, g/dl, mean ± SD	143.6 ± 12.7	145.26 ± 20.5	117 ± 22.8

*Pre* Prednisone, *MMF* Mycophenolate mofetil

Unsurprisingly, the mean hemoglobin level of Group 3 was much lower than that of both Gourp1 and Group2.

### Diffusional Kurtosis imaging

**Fig. 2:**
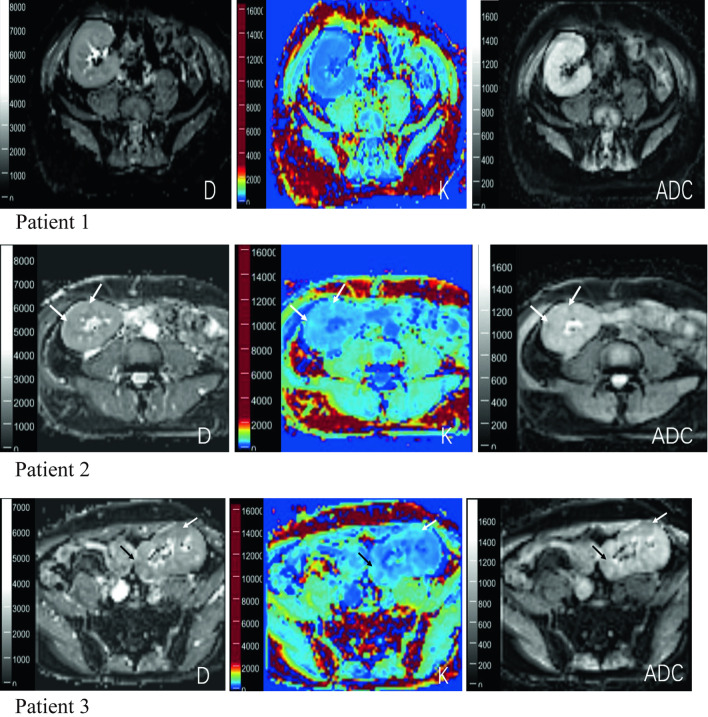
The Diffusion kurtosis Imaging parameter maps (ADC map, K map, and D map) of three patients with different eGFR levels. Patient 1, eGFR = 71.57. The D, K, and ADC map showed that the renal allograft parenchyma had evident signal. Patient 2, eGFR = 47.48. The D and ADC map showed multiple little patchy hypointensive signals in the renal allograft parenchuma, while the K map showed multiple little patchy hyperintensive signals. (as depicted by the arrows). Patient 3, eGFR = 21.49. The D and ADC map showed diffuse low signal intensity in the renal allograft parenchuma, while the K map showed diffuse high signal intensity. (as depicted by the arrows)

The corticomedullary difference is displayed by diffusional kurtosis imaging. (Fig. 2).

Table 2 demonstrated the value of different cortical and medullary DKI parameters in three groups. Except for the mean D of the medulla of group 3 (*P* = 0.277), the renal cortex parameters (including MK and MD values) of the subjects in all three groups were significantly higher than the medulla of the subjects (*P* < 0.05). Nevertheless, the cortical ADC was lower than medullary ADC (*P* < 0.05). ADC and Mean D values of the renal cortex and medulla decreased gradually with the increase of creatinine. In contrast, the Mean K values of the renal cortex and medulla increased gradually with creatinine.

**Table 2:** Comparison of diffusional kurtosis parameters of renal cortex and medulla in each group

	ADC (10^–3^ mm^2^/s)		Kurtosis (10^–3^ mm^2^/s)		D (10^–3^ mm^2^/s)	
	Cortex	Medulla	Cortex	Medulla	Cortex	Medulla
Group 1	1.192 ± 0.049	1.236 ± 0.063	0.470 ± 0.013	0.458 ± 0.017	2.778 ± 0.409	2.625 ± 0.377
	(*P* = 0.000)		(*P* = 0.039)		(*P* = 0.000)	
Group 2	1.141 ± 0.097	1.155 ± 0.116	0.521 ± 0.044	0.511 ± 0.041	2.585 ± 0.237	2.533 ± 0.230
	(*P* = 0.25)		(*P* = 0.000)		(*P* = 0.001)	
Group 3	1.016 ± 0.116	1.049 ± 0.105	0.554 ± 0.042	0.535 ± 0.039	2.183 ± 0.323	2.178 ± 0.197
	(*P* = 0.002)		(*P* = 0.000)		(*P* = 0.27)	

Statistical analysis was employed using the t-test. Data presented as mean ± SD. A *P* < 0.05 was considered statistically significant

**Fig. 3:**
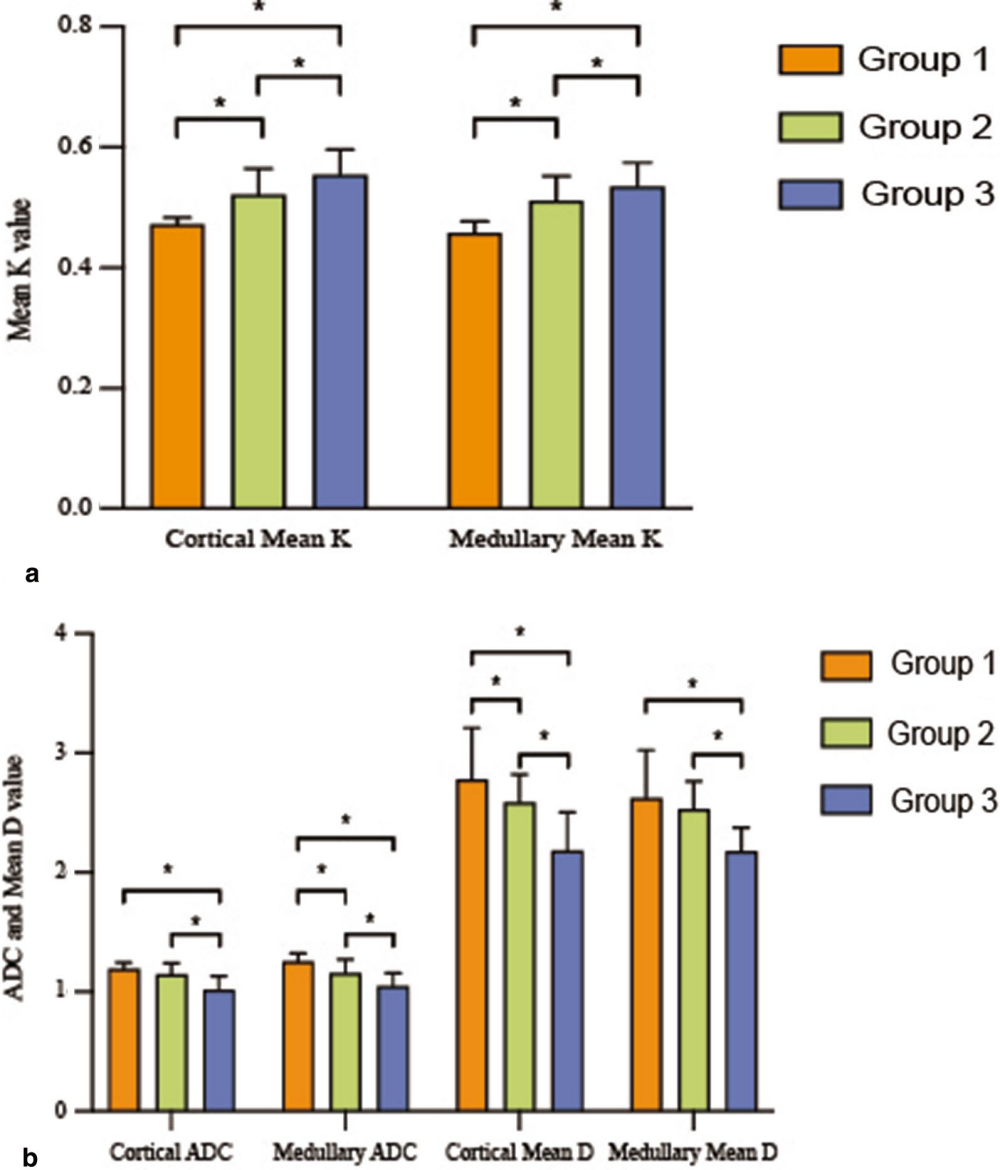
Comparisons of cortical and medullary diffusion kurtosis parameters between different groups. **a** Cortical and Medullary Mean K differ significantly among eGFR levels (*P* < 0.001). **b** ADC and Mean D of both cortex and Medulla differ significantly among eGFR levels (*P* < 0.001), except Cortical ADC and Medullary Mean D between Group 1 and Group 2

As showed in Fig. 3, all parameters showed significant differences (*P* < 0.05) except Cortical ADC and Medullary Mean D between Group 1 and Group 2 (*P* = 0.117, *P* = 0.257, respectively).

### Correlations of DKI parameters with eGFR

**Fig. 4:**
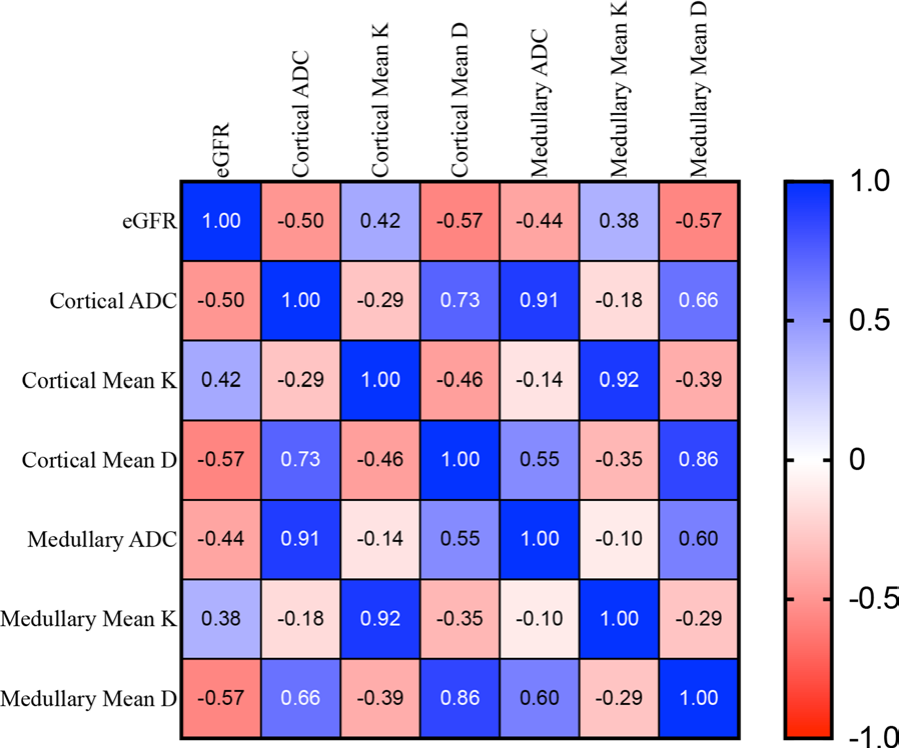
Correlation analysis results between eGFR and diffusion kurtosis parameters (*P* < 0.05)

The Mean K of renal cortex and medulla of all the patients showed positive correlations with eGFR (r = 0.42, 0.38; *P* < 0.001), while the ADC and MD of renal cortex and medulla all correlated negatively with eGFR (r = − 0.49, − 0.44, − 0.57, − 0.57, respectively; *P* < 0.001). (Fig. 4).

### ROC analysis results

**Fig. 5:**
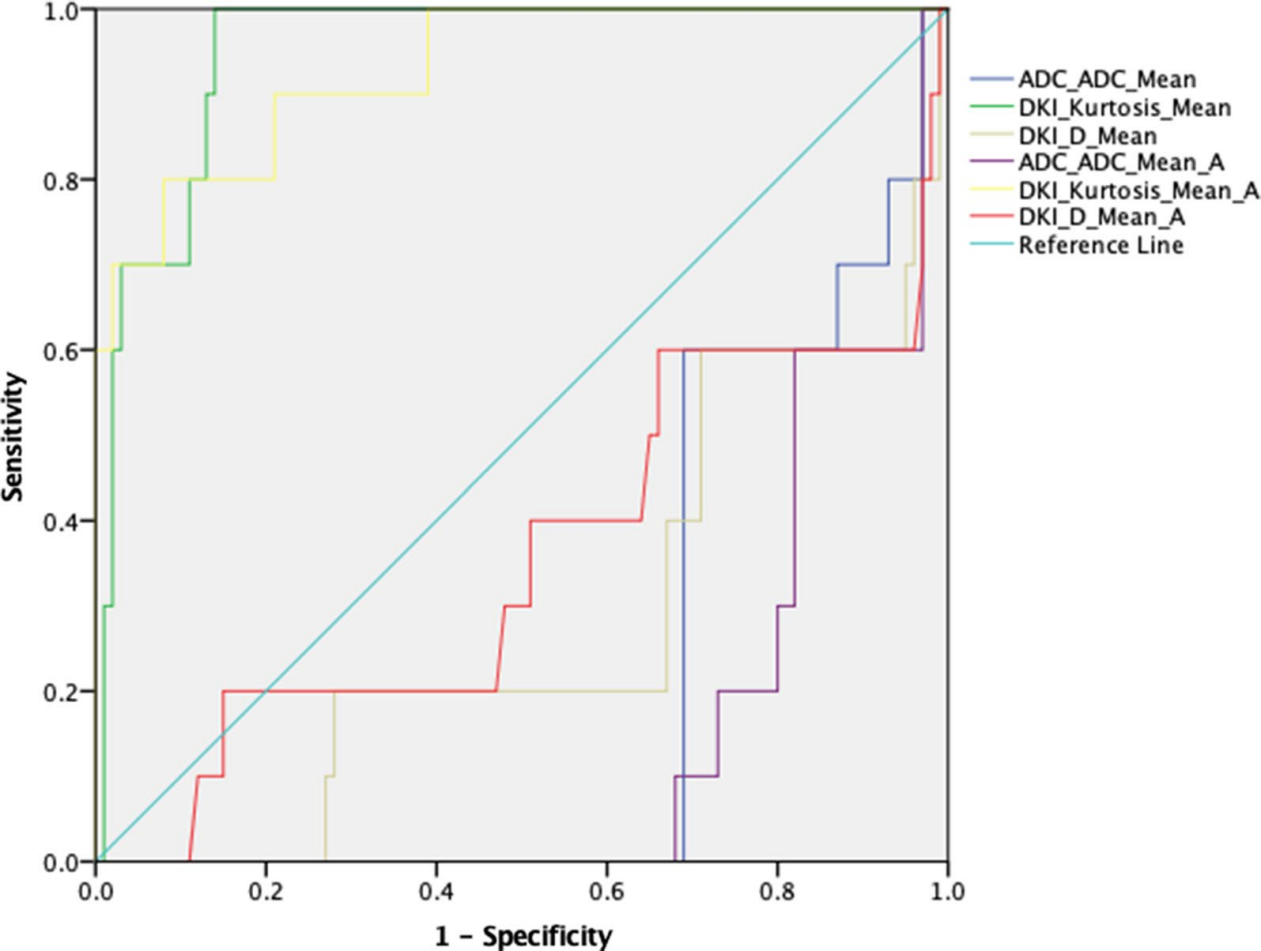
Receiver operating characteristic curves of diffusion kurtosis parameters

In each of these six parameters, the Mean K of the renal cortex showed the most prominent area under the stove of 0.967. In contrast, the medullary Mean K showed a comparable area under the curve of 0.960. When 0.491 was set as the cutoff value, cortical mean K's sensitivity to predicting normal and mild impaired renal function is 87%, and the specificity is 100%. Meanwhile, When 0.499 was set as the cutoff value, the medullary Mean D demonstrated a sensitivity of 61% and specificity of 100% for predicting normal and mildly impaired renal function. (Fig. 5 and Table 3).

**Table 3:** Performances of diffusion kurtosis imaging parameters in predicting decreased transplanted kidney function

Index	ADC (10^–3^ mm^2^/s)		Kurtosis (10^–3^ mm^2^/s)		D (10^–3^ mm^2^/s)	
	Cortex	Medulla	Cortex	Medulla	Cortex	Medulla
AUC	0.218	0.142	0.967	0.960	0.282	0.344
Cut off value		1.429	0.491	0.499		
Sensitivity%		100%	87%	61%		
Specificity%		3%	100%	100%		
*P* value	(*P* = 0.051)	(*P* = 0.042)	(*P* = < 0.022)	(*P* = < 0.041)	(*P* = 0.085)	(*P* = 0.104)

### The results of inter-reader agreement assessment

The results of the ICCs were displayed in the Additional file 1: Table 1. The results of ICCs ranged from 0.83 to 0.99, representing fair agreements for the measurement data.

### Pathology findings

**Fig. 6:**
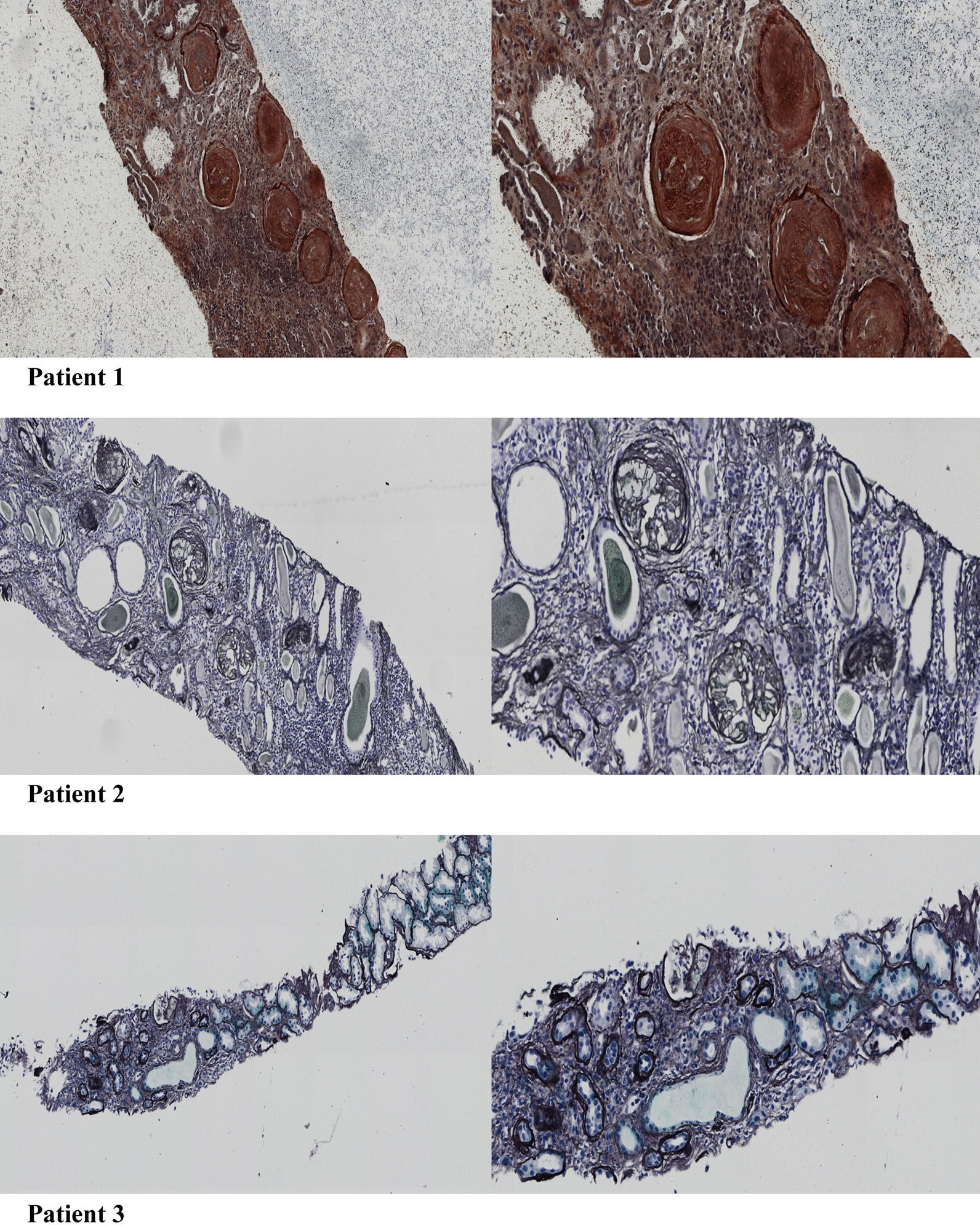
Pathological images of three patients with different eGFR levels renal pathology (PASM *20and *40)

We randomly selected five patients from group 2 to confirm the diagnose of CAI. The five patients' primary pathologic findings were glomerulosclerosis and tubular atrophy/interstitial fibrosis, which conform to CAI diagnosis (Fig. 6).

## Discussion

The present study first proposed the non-Gaussian DKI model as a potential non-invasive method for evaluating CAI.

Several new advances in MRI have enabled the non-invasive assessment of allograft. As reviewed by Ljimani et al. [7], with the development of MRI, multiple of MRI parameters showed the potential of assessment of allograft function. As first investigated in dynamic contrast-enhanced (DCE) [16],parameters likeDWI [17, 18], DTI [6, 19, 20], ASL [21, 22], BOLD [23, 24] then were also used for allograft renal imaging to assess the allograft function. Jensen et al. first proposed DKI in 2005 [25], as an extension of conventional DWI, which requires ultrahigh b-values (> 1000 s/mm^2^) and a modified image post-processing procedure. The traditional model of DWI was established based on the assumption that water diffusion exhibits Gaussian behavior without any restriction and that the diffusion-weighted MRI signal mono-exponentially decreases with increasing b-values; however, a deviation from simple mono-exponential decay is readily identified in the kidney, under either healthy or pathological conditions [26]. DKI could be used to investugate non-Gaussian diffusion of water with a polynomial model and has been used to identify the heterogeneity of cellularity and microstructural complexity [27, 28].

DKI can yield two characteristic variables: D and K. D is the diffusion coefficient corrected by a non-Gaussian bias, and K quantifies the deviation of tissue diffusion from a Gaussian pattern [25, 28]. Recently, in animal models, DKI has been used to assess liver fibrosis [29, 30], which demonstrated additional meaningful information different from that of conventional DWI. Only two studies focused on DKI in healthy kidneys, which showed conflicting results [31, 32]. Furthermore, the findings revealed that the question of whether the maximal b- values (600 and 1000 s/mm) are sufficiently high remains debatable. Huang et al. [31] showed that in a normally functioning kidney, the cortex's MK value is lower than that of the medulla. Among these diffusion kurtosis indicators, the difference between cortex and medulla is reliable with the presence of radially-oriented vessels, tubules, and collecting ducts in the medulla [31]. Interestingly, Pentang et al. [32] showed that the cortical MK is larger than the medullary MK.

As a particular metric of the DKI model, K has been hypothesized to represent the direct interaction of water molecules with the cell membrane intracellular compounds, and expanded K recommends that it has increasingly irregular and heterogeneous environments with numerous great interfaces. In tumor cells, an increased nuclear-cytoplasmic ratio and microstructural were revealed by K value [27, 33]. The precise basic meaning of diffusional kurtosis metrics has yet been grasped, and DKI acquisition has not yet been perfected.

Liu et al. [34] found that in the pathogenesis of IgAN, the progressive loss of glomerular capillary structures and the disappearance of glomerular cellular elements with replacement by an expanding extracellular matrix and fibrous tissue could result in more complex microstructure and marked variation in cell size and shape than in healthy kidneys, leading to increased K.

Our study found that the MK increased gradually with the deterioration of kidney function, which indicates the increase of a much more irregular and heterogeneous environment in renal allograft with the worsening of renal function. As we know, the primary pathology change of CAI is glomerulosclerosis and tubular atrophy/interstitial fibrosis, which means a tendency of the more irregular and heterogeneous environment in the renal allograft.

Liu et al. also demonstrated that K showed better performance than ADC in glomerulosclerosis in terms of diagnostic efficacy, with a relatively larger AUC and stronger correlation. However, the level of statistical significance was not achieved [34]. These results indicate that the K in the DKI model showed clinical potential for assessing the severity of renal sclerosis in the glomeruli and providing more information than ADC.

Our study found that ADC and the Mean D value of cortex in patients with severely decreased eGFR were significantly lower than those in higher eGFR. In comparison, the Mean K value in patients with higher eGFR was lower than in patients with severely reduced eGFR.

According to our research, although all the six parameters showed significant differences except cortical ADC and medullary Mean D between Group 1 and Group 2, the ROC's considerable differences were only found in ADC of Medulla and MK of cortex and medulla. However, the ADC demonstrated an extremely low specificity, and the MK of the cortex showed the largest AUC. Meanwhile, we performed a random autopsy to confirm the histography change of renal allograft. We found glomerulosclerosis and tubular atrophy/interstitial fibrosis of the randomly selected patients, which demonstrated that K increased with the deterioration of renal function and renal fibrosis progression. We suggested that Mean K showed excellent CAI prediction for identifying both glomerulosclerosis and tubular atrophy/interstitial fibrosis.

Simultaneously, the higher Mean K value of patients with decreased renal function may be partially due to interstitial fibrosis. The higher cell density and collagen deposition may result in lower ADC values in renal allografts [35]. This characteristic showed that DKI parameters have broad clinical application prospects in the non-invasive screening of renal allografts' function at various stages. The Mean K in the cortex had a sensitivity of 87% and a specificity of 100% when 0.491 was used as the cutoff value for predicting impaired allograft activity..

There are limitations to this study. First, the number of patients with normal eGFR was small. Moreover, it limits the accuracy of ROC curves. Second, not all the patients were performed biopsy in this study to analyze the quantitative correlation between histopathologic results and DKI parameters.

So, although we confirmed that the DKI model was associated with the changes of eGFR and can assess CAI to some extent, if the DKI model can evaluate CAI evolution more accurately before the change of eGFR, it still needs to be studied. In the future, we aim to perform a more extensive sample size research to explore if the micro-changes detected by the DKI model can stand for the change of CAI more accurately, even earlier than eGFR.

## Conclusions

In conclusion, the non-invasive KI model was closely associated with the eGFR as allograft injury progresses, as renal perfusion might be reduced. The parameter MK of the renal cortex can non-invasively assess CAI to some extent. The DKI technique is correlated with eGFR and can be expected to be a non-invasive method to evaluate CAI potentially.

## Supplementary Information

**Additional file 1: Table 1**. The ICCs of two observers on multiple parameters.

## Data Availability

All the data and materials can be offered if required. Xin Zheng (mike.zheng@163.com) can be contacted for the requesting of data from this study.

## Abbreviations

ADC: Apparent diffusion coefficient
MK: Mean kurtosis
MD: Mean diffusivity
DTI: Diffusion tensor imaging
eGFR: Estimated glomerular filtration rate
CAI: Chronic allograft injury
MRI: Magnetic resonance imaging
ROC: Receiver operating characteristic
