# Supplementary material for: Assessment of chronic allograft injury in renal transplantation using diffusional kurtosis imaging
Source: BMC Med Imaging. 2021 Apr 7;21:63. doi: 10.1186/s12880-021-00595-3 (PMC8028790; doi:10.1186/s12880-021-00595-3)
Supplement: Supplementary file 1 — Additional file 1: Table 1. The ICCs of two observers on multiple parameters. [file 12880_2021_595_MOESM1_ESM.docx]

**Supplementary materials**

| **Table 1: The ICCs of two observers on multiple parameters** | | | | | | | |
| --- | --- | --- | --- | --- | --- | --- | --- |
|  |  |  | Cortex | |  | Medulla | |
|  |  |  | ICC | 95% CI |  | ICC | 95% CI |
|  | **ADC** |  | 0.973 | 0.956–0.984 |  | 0.832 | 0.825–0.911 |
|  | **Kurtosis** |  | 0.896 | 0.857–0.934 |  | 0.837 | 0.812–0.871 |
|  | **D** |  | 0.991 | 0.980–0.997 |  | 0.935 | 0.889–0.953 |

ICC, intraclass correlation; CI, confidence intervals.
